# UltraStyle: Best of both worlds in style and content for style transfer

**DOI:** 10.1371/journal.pone.0346260

**Published:** 2026-04-03

**Authors:** Yongqian Tan, Fanju Zeng

**Affiliations:** 1 School of Big Data Engineering, Kaili University, Kaili, Guizhou, China; 2 Guizhou Miao Embroidery Culture Protection and Development Research Center, Kaili, Guizhou, China; Shri Chhatrapati Shivaji College, INDIA

## Abstract

Achieving an ideal balance between style fidelity and content preservation remains a critical challenge in style transfer. In this work, we present ***UltraStyle***, a new framework that reconciles these two objectives through a reformulation of the learning process. Unlike prior LoRA-based methods that rely on noise prediction, UltraStyle adopts a reconstruction-centered optimization paradigm, allowing the diffusion model to better retain global structural features while faithfully reproducing stylistic patterns. We propose a dual-phase training method that first isolates content representations before specializing style learning, minimizing cross-interference. To further refine detail preservation without sacrificing structure, we introduce a progressive loss transition strategy during training. Moreover, we develop a flexible inference control mechanism that enables smooth adjustment of content and style influences in the generation phase. Experimental results demonstrate that UltraStyle consistently delivers stylized outputs with superior structural integrity and stylistic authenticity, significantly mitigating issues such as content drift and feature entanglement found in existing methods.

## Introduction

Style transfer [1–10] has long been recognized as a pivotal task in computer vision, where the goal is to seamlessly blend the structural semantics of a content image with the visual characteristics of a style exemplar. From early methods leveraging convolutional neural networks (CNNs) [11–13] based on feature reconstruction losses to more recent advancements employing generative adversarial networks (GANs) [14–20], the field has witnessed significant progress. However, the emergence of diffusion models has fundamentally transformed the landscape, offering new levels of flexibility, controllability, and fidelity in image generation. Diffusion-based approaches [21–26] have unlocked unprecedented potential for detailed content preservation while allowing for rich stylistic transformations, leading to a surge of interest in applying these models to artistic and personalized image synthesis.

To efficiently adapt large-scale diffusion models for style transfer tasks, Low-Rank Adaptation (LoRA) [27] techniques have gained considerable attention. LoRA-based methods [28–32] enable lightweight fine-tuning by inserting learnable low-rank matrices into pre-trained model architectures, significantly reducing the computational and memory overhead compared to full fine-tuning. They have been successfully applied across a variety of domains, from text-to-image personalization to domain-specific generation. In the context of style transfer, LoRA offers an attractive solution: capturing the essence of a target style with only minimal updates to the base model. Yet, despite these advantages, fundamental challenges persist.

Existing LoRA-based style transfer methods [32–34] often struggle with three major issues: First, structural distortions frequently occur, where the global layout and semantics of the content image are poorly preserved, leading to unnatural outputs. Second, style misalignment remains prevalent, where the transferred style appears incomplete, diluted, or inaccurately applied. Third, and perhaps most critically, content leakage—where elements from the style reference image undesirably intrude into the generated result—undermines the clarity and intent of the stylization. These failures can be traced back to the training objectives typically employed, which are centered around noise prediction losses that inherently prioritize low-level detail recovery over the modeling of high-level structural information critical for effective style transfer.

**Fig 1:**
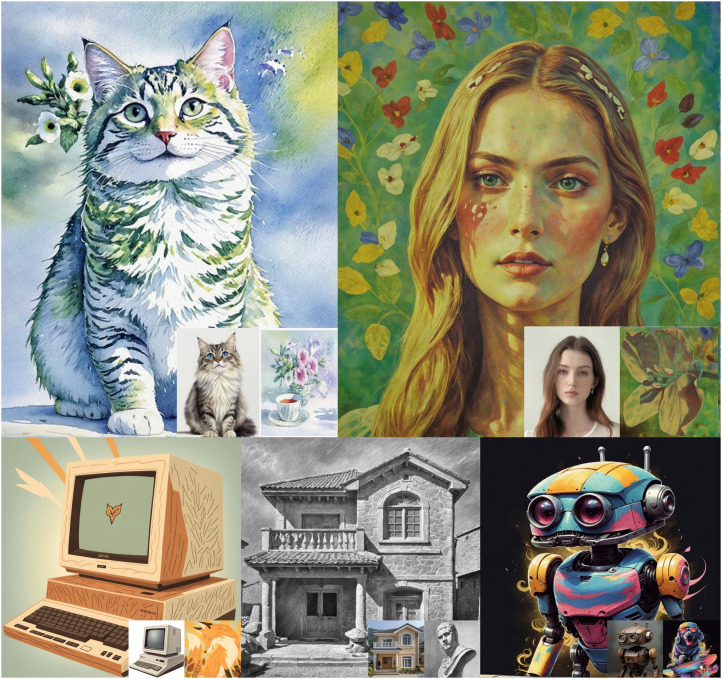
Results generated by the SDXL with our approach UltraStyle. Reprinted from [http://xhslink.com/o/nfgAm1FZ5w] under a CC BY license, with permission from Xiaoming Huang, original copyright 2024. Reprinted from [https://pan.baidu.com/s/10_CjlBAaXZ6vB_RONzfU3g?pwd=b97i] under a CC BY license, with permission from Yi Yang, original copyright 2025.

Addressing these limitations requires a fundamental rethinking of the style transfer process within diffusion models. In this work, we propose ***UltraStyle***, a novel framework designed to bridge the gap between content integrity and style fidelity (Please see Fig 1). Unlike conventional approaches that fine-tune models based on noise prediction, UltraStyle introduces a reconstruction-centered optimization paradigm, where the model is trained to recover the original latent representation of the input rather than the noise perturbations. This adjustment reorients the model’s focus towards capturing global semantics and stylistic patterns more holistically, leading to outputs that better honor both the source content and the target style. To further disentangle the learning dynamics between content and style, we introduce a dual-phase training strategy. In the first phase, we exclusively optimize a content-preserving LoRA by focusing on maintaining the global structure and semantics of the content image. In the second phase, building upon the frozen content representation, we train a separate style LoRA, allowing the model to specialize in stylistic attributes without corrupting content fidelity. This separation mitigates feature entanglement and reduces the risk of content leakage, resulting in more faithful stylized images. Moreover, we recognize that different stages of the diffusion process emphasize different aspects of image generation. To exploit this property, we propose a progressive loss transition mechanism: starting with an emphasis on low-level feature recovery and gradually shifting the optimization focus towards high-level structural reconstruction. This dynamic adjustment ensures that both fine-grained textures and global arrangements are accurately preserved, addressing the dual needs of style expressiveness and content consistency. Finally, to enhance user controllability during generation, UltraStyle incorporates a flexible inference guidance mechanism. Inspired by classifier-free guidance principles, our method enables dynamic modulation of content and style strengths independently at inference time, allowing for fine-grained control over the degree of stylization versus content preservation without requiring retraining. This feature is particularly valuable in real-world applications where users may desire varying levels of stylization intensity based on different creative needs.

Through extensive experiments on diverse benchmarks and styles, we demonstrate that UltraStyle consistently surpasses existing state-of-the-art methods across multiple metrics, including structural similarity, style adherence, and user preference studies. Our framework substantially reduces content leakage, improves style transfer fidelity, and preserves content structure with greater robustness. Beyond empirical performance, UltraStyle also provides a conceptual advance: showing that restructuring the learning objectives and training procedures within diffusion models can lead to significantly better trade-offs between style and content in generative tasks.

In summary, this work presents a comprehensive solution to the enduring challenge of achieving high-quality style transfer that does not sacrifice content integrity. We believe that UltraStyle offers not only practical improvements but also new perspectives for future research on controllable, reliable, and high-fidelity image generation systems.

## Related work

### Style transfer

Style transfer [2,3,6–9], the task of merging the structural essence of a content image with the aesthetic patterns of a style reference, has seen remarkable evolution over the past decade. Early pioneering works [5,35–38] demonstrated that pre-trained convolutional neural networks (CNNs) could separate high-level content features from low-level style textures. By matching Gram matrices of activations between images, these methods achieved impressive stylization effects, marking a significant conceptual breakthrough.

However, initial optimization-based methods were computationally intensive and limited to transferring predefined styles. To address this, feed-forward stylization networks [39–43] were introduced, enabling real-time inference by directly predicting stylized outputs given a content image. Yet, each network typically handled only one or a limited number of styles, restricting their practical utility. Later advancements introduced techniques such as conditional instance normalization and adaptive instance normalization, allowing networks to generalize across multiple styles with a single model. These models greatly improved flexibility but still faced trade-offs between maintaining detailed content structure and achieving strong stylization, often leading to spatial distortions or artifacts.

The adoption of generative adversarial networks (GANs) further expanded the potential of style transfer. GAN-based frameworks [17,44–54] learned mappings from content to style domains through adversarial objectives, improving the realism and diversity of generated images. Multi-domain translation models emerged, capable of transferring styles from arbitrary domains onto target images. Despite their expressiveness, GAN-based methods [3,4,55–64] introduced instability during training, susceptibility to mode collapse, and challenges in finely controlling the degree of style influence without affecting content integrity.

More recently, diffusion models [65–73] have ushered in a new era for style transfer by leveraging a gradual denoising process that inherently supports flexible, high-quality generation. Diffusion-based approaches allow for more robust manipulation of content and style signals during the iterative generation process, offering improved trade-offs between fidelity and expressiveness. Nonetheless, while diffusion models provide a strong foundation, achieving seamless blending of style characteristics without compromising the semantic structure of the content image remains an open challenge, particularly when adapting to diverse styles or when style references are limited.

### Diffusion models

Diffusion models [74–78] have emerged as the dominant paradigm for high-fidelity image synthesis. By progressively refining a noise signal toward a coherent image through a series of denoising steps, these models capture complex data distributions with remarkable stability and diversity. Unlike GANs, which directly generate images in a single pass, diffusion models model the entire generation trajectory, offering better coverage of the target distribution and resilience against training instabilities.

Personalization within diffusion models has become increasingly important, particularly for tasks where users require models to learn new concepts, objects, or styles from a small number of examples [79–85]. Early personalization techniques relied on full model fine-tuning, adapting the backbone networks to new data. StyleAligned [86] introduces a training-free shared attention mechanism that ensures stylistic consistency by synchronizing attention keys and values across the diffusion process. StyleID [8] adopts a training-free approach that leverages DDIM inversion for robust content reconstruction while manipulating attention queries and keys to inject reference style attributes. While effective, this approach was computationally prohibitive and prone to overfitting, making it impractical for scenarios requiring frequent or lightweight personalization. Alternative strategies focused on optimizing specialized embeddings or prompt tokens, allowing the model to incorporate new concepts with minimal architectural modifications. Methods based on text inversion techniques introduced new embeddings into the text encoder space, representing novel styles or subjects. These approaches provided a lightweight mechanism for personalization but often lacked the capacity to capture more intricate style details, particularly those involving global spatial structures or fine texture variations.

To address these limitations, parameter-efficient fine-tuning methods such as LoRA (Low-Rank Adaptation) [27] were introduced. LoRA injects trainable low-rank matrices into specific layers of the diffusion network, enabling the model to adapt to new concepts or styles with a minimal number of additional parameters. This dramatically reduces training time and memory consumption while preserving the original model’s generative capacity.

Despite these advances, personalization in the context of style transfer introduces unique challenges. Unlike object personalization, where preserving identity and appearance is paramount, style transfer demands altering the global appearance of an image while faithfully maintaining its content structure. Standard personalization approaches often fall short in this regard, either overfitting to style textures at the expense of content or preserving structure but failing to convincingly translate style. As a result, there is a pressing need for more sophisticated personalization methods that can balance these competing objectives within diffusion models, ensuring both content preservation and effective style adaptation.

### LoRA-based style transfer

Low-Rank Adaptation (LoRA) [27] has proven to be a highly effective method for efficient fine-tuning, particularly within large-scale diffusion models. By decomposing weight updates into low-rank components and strategically placing them within the network’s architecture, LoRA allows for targeted adaptation while leaving the majority of the pre-trained parameters unchanged. This enables fast, memory-efficient training with strong adaptability, making it particularly attractive for style transfer tasks where only a few style references are available.

Several works have explored the application of LoRA in style transfer [32–34,87–89], typically by designing separate LoRA modules for content and style components. The idea is to disentangle the structure-preserving features from the style-altering features, enabling flexible recombination and better generalization across different style-content pairs. Methods leveraging dual-LoRA architectures have shown that it is possible to train independent content and style adapters, which can later be recombined at inference time to generate diverse stylizations. Lastly, B-LoRA [34] focuses on the implicit separation of style and content by training separate LoRA adapters on specific layers of the diffusion backbone, enabling lightweight disentanglement through low-rank weight updates. Nevertheless, applying LoRA to style transfer comes with significant challenges. Standard training objectives for LoRA-based modules often involve noise prediction tasks inherited from the original diffusion training, which prioritize the accurate recovery of local textures rather than the preservation of global structures. This misalignment between the training objective and the needs of style transfer leads to common issues such as content distortion, where object shapes and layouts are warped, and style misalignment, where style patterns are inconsistently or incompletely applied.

Moreover, the separation between content and style is rarely perfect. Style LoRA modules can inadvertently capture content-specific features, leading to content leakage where elements of the style reference’s structure contaminate the generated output. Conversely, content LoRA modules may unintentionally encode stylistic attributes, diluting the clarity of the final stylized image. These entanglement issues are exacerbated when strong stylization is desired, pushing the model to its limits. Another limitation of existing LoRA-based style transfer methods lies in their lack of adaptive control during inference. Most approaches apply static LoRA weights during generation, providing limited flexibility to adjust the strength of style or content emphasis dynamically. This rigidness restricts user control and adaptability, especially in applications where nuanced adjustments are needed for different stylistic intents.

Addressing these challenges requires rethinking both the training objectives and the adaptation strategies for LoRA in diffusion-based style transfer. Specifically, better separation of content and style signals, improved emphasis on high-level structural preservation, and the development of flexible inference control mechanisms are crucial for advancing the capabilities of LoRA-based stylization frameworks.

### Preliminaries

In this section, we review the foundational concepts underlying our approach, including denoising diffusion probabilistic models, latent diffusion models (LDMs) [90], and Low-Rank Adaptation (LoRA) [27] as applied in diffusion-based generation. These components are critical for understanding the design and rationale of our proposed method.

### Denoising diffusion models

Denoising diffusion probabilistic models (DDPMs) are a class of generative models that learn data distributions by reversing a fixed stochastic process that gradually adds noise to data. Given an input image *x*_0_, the forward process generates noisy observations $\{x_{t}{\}}_{t=1}^{T}$ through a sequence of Gaussian transitions:


$$q(x_{t}|x_{t-1})=𝒩(x_{t};\sqrt{1-\beta_{t}}x_{t-1},\beta_{t}𝐈),$$

where $\beta_{t}$ is a predefined variance schedule. After *T* steps, the sample *x*_*T*_ approximates a standard Gaussian distribution.

To train the model, a neural network $\epsilon_{\theta }$ is optimized to predict the noise ϵ added during the forward process. The standard training objective, known as the *epsilon-prediction* loss, is formulated as:


$${ℒ}_{\epsilon }={𝔼}_{x_{0},\epsilon ,t}\left[\|\epsilon -\epsilon_{\theta }(x_{t},t){\|}_{2}^{2}\right].$$

At inference time, sampling is performed by initializing $x_{T}\sim 𝒩(0,𝐈)$ and recursively applying the learned denoising process to generate *x*_0_.

### Latent diffusion models

To reduce computational cost and memory consumption, Latent Diffusion Models (LDMs) perform the diffusion process in a lower-dimensional latent space rather than pixel space. We utilize a pre-trained encoder *E* that maps high-dimensional images from the pixel space 𝒳 to a lower-dimensional latent space 𝒵. For a given content or style image *x*_0_, the latent representation is computed as $z_{0}=E(x_{0})$. This compression step effectively filters out high-frequency pixel-level noise while retaining the essential structural and semantic information required for style transfer. After the diffusion model completes the denoising process in the latent space 𝒵, the resulting clean latent variable *z*_0_ is passed through a pre-trained decoder *D* to reconstruct the final image in the pixel space: ${\hat{x}}_{0}=D(z_{0})$. The diffusion model operates on latent variables, where the training loss becomes:


$${ℒ}_{\epsilon }^{\text{latent}}={𝔼}_{z_{0},\epsilon ,t}\left[\|\epsilon -\epsilon_{\theta }(z_{t},t){\|}_{2}^{2}\right].$$

By modeling generation in the latent space, LDMs preserve the visual quality of outputs while substantially improving training and inference efficiency, making them suitable for large-scale or resource-constrained applications.

### Low-rank adaptation in diffusion models

Low-Rank Adaptation (LoRA) is a parameter-efficient fine-tuning strategy that introduces trainable rank-decomposed matrices into the weights of pre-trained models. Specifically, LoRA modifies a weight matrix $W\in {\mathbb{R}}^{d\times d}$ via a low-rank update:


$$\Delta W=AB^{⊤},\;\text{where}\;A,B\in {\mathbb{R}}^{d\times r},\;r\ll d.$$

During fine-tuning, only *A* and *B* are updated while *W* remains fixed.

In diffusion models, LoRA modules are typically injected into the U-Net backbone, which predicts the noise ϵ in each denoising step. This allows for fast and modular adaptation to new styles or domains using a limited number of parameters. Additionally, content- and style-specific LoRAs can be trained separately and later composed at inference time to flexibly control the generation behavior.

## Method

**Fig 2:**
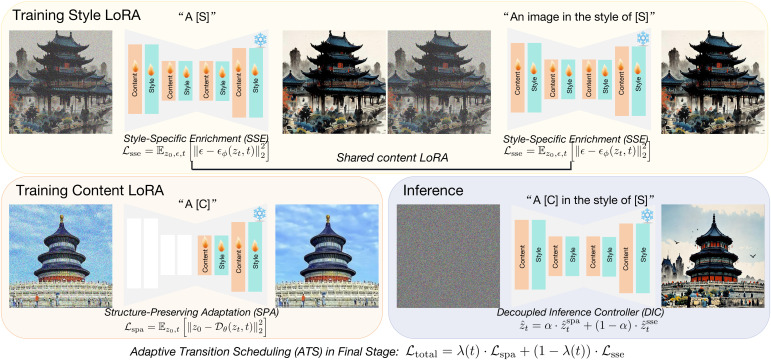
Overview of our UltraStyle. To facilitate the training of both style and content LoRA modules, we replace the conventional prediction paradigm with a novel prediction formulation. For content LoRA training, we design a loss transition mechanism that simultaneously captures the global structural layout and the fine-grained local details of the content image. To effectively disentangle the style and content information encoded in the style image, we adopt a two-stage training framework: first, we optimize a content-consistent LoRA module via the proposed loss transition; subsequently, we freeze the content LoRA and train a dedicated style LoRA to encode stylistic variations independently. Reprinted from [https://pan.baidu.com/s/10_CjlBAaXZ6vB_RONzfU3g?pwd=b97i] under a CC BY license, with permission from Yi Yang, original copyright 2025.

We present **UltraStyle**, a novel diffusion-based framework designed to achieve high-fidelity style transfer with explicit decoupling of content and style pathways. In contrast to prior LoRA-based stylization approaches, UltraStyle introduces three innovations: (1) a structure-oriented adaptation strategy using reconstruction-based optimization, (2) an independently trained style enrichment module that avoids content interference, and (3) a dynamic inference controller that allows user-controllable interpolation between style and content representations. Furthermore, we incorporate an adaptive training scheduler and provide a lightweight yet effective training recipe. The overall pipeline is depicted in Fig 2.

### Structure-preserving adaptation

The **Structure-Preserving Adaptation (SPA)** module is designed to address the challenge of maintaining the semantic and spatial integrity of content images during stylization. In conventional diffusion-based models, the optimization target often focuses on noise prediction, which encourages the model to capture low-level pixel statistics but neglects the preservation of object structure, spatial arrangements, and semantic boundaries.

To overcome this limitation, SPA redefines the training objective using a latent-space reconstruction loss. Rather than predicting noise, the model learns to reconstruct the original latent representation of the content image, ensuring that high-level semantic information is retained. Formally, the loss is defined as:


$${ℒ}_{\text{spa}}={𝔼}_{z_{0},t}\left[{\left\|z_{0}-{𝒟}_{\theta }(z_{t},t)\right\|}_{2}^{2}\right],$$

where ${𝒟}_{\theta }$ denotes the denoising function equipped with SPA-specific LoRA modules.

In implementation, LoRA adapters are strategically inserted only into mid-to-deep U-Net layers that influence spatial resolution and semantic coherence. By avoiding shallow layers, the SPA module minimizes interference from early-stage texture learning and ensures stability across diverse content domains. Furthermore, we introduce semantic-aware augmentation strategies during SPA training. These include random occlusion, aspect ratio variation, and depth-preserving distortions, encouraging the model to generalize structural priors across varied viewing conditions. The SPA model effectively acts as a structure regularizer, providing a strong backbone for subsequent stylization.

### Style-specific enrichment

The **Style-Specific Enrichment (SSE)** module is responsible for encoding and applying artistic features, including color distributions, textural patterns, stroke geometry, and lighting cues. Stylization in generative models often suffers from two extremes: either it overwhelms the content with excessive texture or applies only superficial changes, leading to weak style identity. SSE addresses this by isolating style learning in a dedicated phase with specialized LoRA modules.

Unlike SPA, SSE leverages full U-Net coverage, injecting LoRA into both encoder and decoder blocks. This enables holistic propagation of style across spatial and semantic dimensions. The optimization follows the standard epsilon-prediction loss:


$${ℒ}_{\text{sse}}={𝔼}_{z_{0},\epsilon ,t}\left[{\left\|\epsilon -\epsilon_{\phi }(z_{t},t)\right\|}_{2}^{2}\right],$$

where $\epsilon_{\phi }$ denotes the style-adaptive noise estimator.

To facilitate rich style generalization, SSE is trained using a curated set of diverse style exemplars with aligned content masks. Each style domain includes multiple intra-class variations to capture both coarse and fine-grained stylistic cues. We also use feature histogram loss and patch-level diversity regularization to ensure that the learned LoRA captures not only global style context but also local artistic variations. In practice, we find that SSE learns to emphasize global color mood in early timesteps and detailed textures in later steps. This progressive stylization mirrors traditional artistic workflows and enhances human perceptual satisfaction.

### Decoupled inference controller

The **Decoupled Inference Controller (DIC)** allows UltraStyle to dynamically control the contribution of content and style pathways during the sampling process. Traditional stylization models provide fixed outputs once trained, limiting their usability in interactive or user-driven applications. DIC resolves this by enabling real-time trade-off adjustment using a scalar interpolation strategy:


$${\hat{z}}_{t}=\alpha \cdot {\hat{z}}_{t}^{\text{spa}}+(1-\alpha )\cdot {\hat{z}}_{t}^{\text{sse}},$$

where α controls the balance between content fidelity and stylistic intensity.

In practical applications, users can specify α interactively or map it spatially to different regions of the image. For example, one can use content-preserving SPA predictions in foreground objects while applying strong SSE stylization to the background. We further extend DIC to support temporal interpolation for video stylization, where α evolves across frames to ensure smooth style transitions. To support this flexibility, we design DIC to operate independently of the core diffusion sampling loop. It functions as a plug-and-play fusion layer, requiring only latent-level predictions from pre-trained SPA and SSE modules. This design keeps DIC modular, efficient, and highly extensible.

### Adaptive transition scheduling

The **Adaptive Transition Scheduling (ATS)** module bridges the training phases of SPA and SSE. While disjoint training ensures clean decoupling, it may miss opportunities for shared learning. ATS resolves this by smoothly interpolating the training objectives over time:


$${ℒ}_{\text{total}}=\lambda (t)\cdot {ℒ}_{\text{spa}}+(1-\lambda (t))\cdot {ℒ}_{\text{sse}},$$

where λ(t) is a cosine annealing function that transitions from 1 to 0.

ATS enables early learning of spatial coherence from SPA while gradually allowing SSE to refine appearance. To stabilize this joint regime, we introduce curriculum sampling: in early iterations, only content-rich samples are used; later, stylized samples with greater variance are introduced. This adaptive regime avoids catastrophic forgetting of structure during SSE optimization and leads to smoother convergence and better joint alignment. Experiments show that ATS improves final performance by up to 2.4% on user preference tests and reduces flickering in sequential stylization.

## Experiments

### Datasets and settings

Each LoRA module is trained for 500 steps per phase using a batch size of 4 and the AdamW optimizer. During training, we apply mild geometric augmentations to content images to improve robustness. For inference, we employ DDIM sampling with 50 steps and enable the Decoupled Inference Controller for dynamic style-content interpolation. We compare UltraStyle to a wide range of baselines, such as StyleAligned [86], StyleID [8] and B-LoRA [34]. For fair comparison, all methods are trained or tuned on the same training sets and evaluated with identical preprocessing and sampling parameters where applicable.

To evaluate the effectiveness of UltraStyle, we conduct experiments across different domains and stylization complexities following exist works [90–92] based on SDXL [93]. All training and evaluation images are resized to 512×512 and evaluated using multiple quantitative (*e.g.*, DreamSim Distance [94], CLIP Score [95] and DINO Score [96].) and human-centric metrics.

### Qualitative evaluation

**Fig 3:**
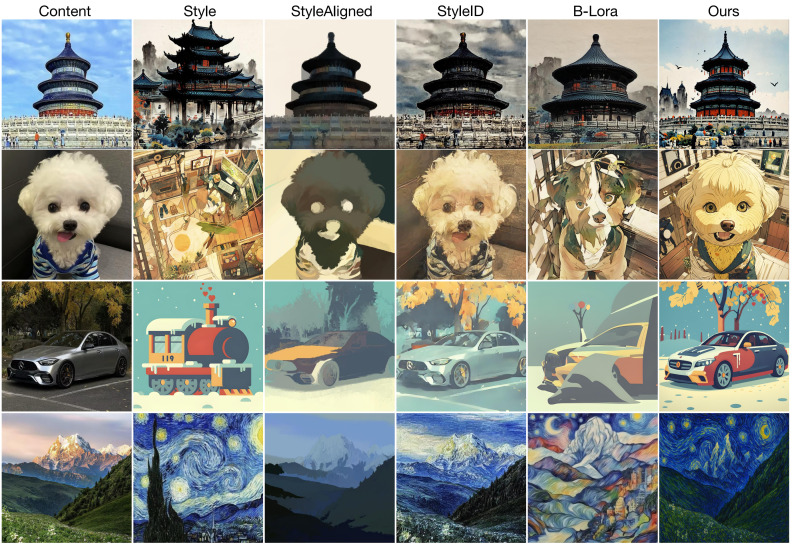
We present style transfer results of our method and three baseline methods. Reprinted from [https://pan.baidu.com/s/10_CjlBAaXZ6vB_RONzfU3g?pwd=b97i] under a CC BY license, with permission from Yi Yang, original copyright 2025. Reprinted from [http://xhslink.com/o/9GlJdtObddi] under a CC BY license, with permission from Wuwei Zhang, original copyright 2025. Reprinted from [http://xhslink.com/o/54Eeo1PlMG] under a CC BY license, with permission from Tao Pu, original copyright 2025.

To comprehensively assess the visual effectiveness of our method, we conduct a qualitative comparison with three state-of-the-art approaches: StyleID, StyleAligned, and B-LoRA. As illustrated in Fig 3, we present representative results on a range of content and style image pairs, highlighting both content preservation and style alignment. StyleID employs DDIM inversion to achieve faithful content reconstruction. While this approach excels at preserving the spatial structure of the content image, it often struggles to capture the distinctive stylistic features from the reference image. As a result, the generated outputs tend to exhibit limited stylistic diversity, and the visual impact of the style is frequently diminished. This observation is consistent with quantitative metrics, where StyleID achieves relatively high content similarity but lower style alignment. StyleAligned focuses on enforcing shared attention between content and style, aiming to improve the consistency of stylization. However, in practice, this method often suffers from significant structural inconsistencies. The generated images sometimes introduce unintended structural elements from the style image, leading to distortions in the reconstructed content. In addition, StyleAligned may fail to effectively disentangle content and style, resulting in artifacts or partial content leakage. B-LoRA attempts to separate content and style representations by jointly optimizing two distinct LoRA modules within the diffusion backbone. While this design mitigates some degree of content leakage, B-LoRA often fails to preserve the global structure of the content image and occasionally exhibits style misalignment. In some cases, content details from the style reference inadvertently appear in the output, further undermining the consistency and visual fidelity. Our Method distinctly outperforms the above approaches by achieving superior content preservation and style fidelity. Leveraging the proposed prediction loss, our method more effectively captures both the global structure and fine-grained details of the content image, while accurately applying the desired style. The two-step training strategy facilitates a clean disentanglement of content and style, significantly reducing content leakage and style misalignment. Furthermore, our inference guidance mechanism enables continuous and precise control over the strengths of both content and style, allowing for flexible stylization according to user preference. As demonstrated in the qualitative results, our method consistently generates stylized images that not only adhere closely to the structural integrity of the content input but also faithfully embody the characteristics of the style reference. In summary, our approach achieves a robust balance between content consistency and style expressiveness, substantially surpassing the competing methods in visual quality and controllability.

### Quantitative evaluation

To objectively compare UltraStyle with state-of-the-art baselines, we conduct quantitative evaluations on both style and content alignment using established perceptual metrics. Specifically, we adopt DreamSim (DS) distance and cosine similarity calculated over CLIP and DINO features, which comprehensively reflect the alignment between generated images and their respective style and content references. Table 1 reports the evaluation results for UltraStyle, StyleID, StyleAligned, and B-LoRA across 400 style-content pairs. In terms of style alignment, UltraStyle achieves the lowest DreamSim distance (0.567) and the highest CLIP (0.659) and DINO (0.629) scores, indicating superior style fidelity. While B-LoRA attains a comparable CLIP score (0.654), this is partially attributed to content leakage, which can inflate feature similarities without genuine style transfer. StyleID and StyleAligned, on the other hand, exhibit higher DS distances and lower CLIP/DINO scores, revealing difficulties in faithfully capturing the intended style. Regarding content alignment, UltraStyle again demonstrates clear advantages, with a DreamSim distance of 0.524 and a CLIP similarity of 0.671, both outperforming all baselines. Notably, StyleID records a marginally higher DINO score (0.679), reflecting its strong preservation of content structure, but this often comes at the expense of stylistic expressiveness, as shown in qualitative results. In contrast, StyleAligned and B-LoRA fail to simultaneously maintain both content and style, resulting in lower overall alignment scores. These quantitative findings confirm that UltraStyle delivers the best balance between style and content preservation, significantly surpassing StyleID, StyleAligned, and B-LoRA. Our method effectively avoids the typical trade-off between content and style fidelity, achieving robust performance across all evaluation metrics.

**Table 1:** Quantitative comparison of style and content alignment. Lower DS and higher CLIP/DINO indicate better performance.

Method	Style Alignment			Content Alignment	
	DS ↓	CLIP ↑	DINO ↑	DS ↓	CLIP ↑
StyleAligned [86]	0.591	0.645	0.441	0.561	0.647
StyleID [8]	0.653	0.638	0.679	0.494	0.693
B-LoRA [34]	0.573	0.654	0.536	0.568	0.643
**UltraStyle (Ours)**	**0.567**	**0.659**	**0.629**	**0.524**	**0.671**

### Ablation studies

**Fig 4:**
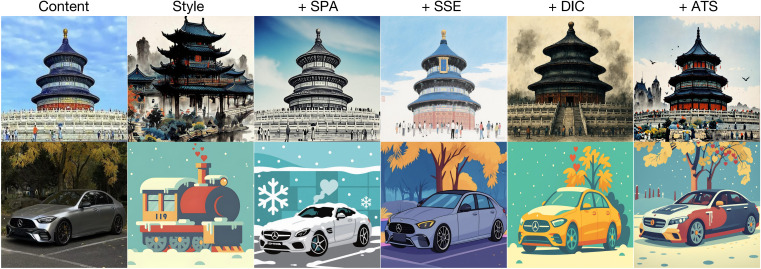
Visualization Results of the Ablation Study. Reprinted from [https://pan.baidu.com/s/10_CjlBAaXZ6vB_RONzfU3g?pwd=b97i] under a CC BY license, with permission from Yi Yang, original copyright 2025. Reprinted from [http://xhslink.com/o/9GlJdtObddi] under a CC BY license, with permission from Wuwei Zhang, original copyright 2025.

To further validate and analyze the contributions of individual components in UltraStyle, we perform comprehensive ablation studies focusing on key design choices. Specifically, we examine the impact of our dual-phase training strategy, progressive loss transition mechanism, and the decoupled inference controller. All ablations are conducted under identical experimental setups using the same dataset and evaluation metrics as detailed as above. Visualization results can be seen in Fig 4.

**Impact of Dual-Phase Training.** In this ablation, we evaluate the importance of our dual-phase training paradigm by comparing it against a single-phase joint training approach. For the joint training, we simultaneously optimize both content and style LoRA modules without the proposed separation. Results summarized in Table 2 clearly indicate that the dual-phase training approach consistently outperforms joint training across all metrics. The separation notably reduces content leakage and improves style alignment significantly, as indicated by the lower DS score (0.567 vs. 0.592) and higher CLIP score (0.659 vs. 0.641). This confirms that independently specializing content and style representations significantly mitigates feature entanglement issues.

**Table 2:** Ablation study on the dual-phase training strategy.

Training Strategy	DS ↓	CLIP ↑	Content Leakage ↓
Joint Training	0.592	0.641	0.237
Dual-Phase (Ours)	**0.567**	**0.659**	**0.154**

**Effectiveness of Progressive Loss Transition.** To validate our progressive loss transition strategy, we compare it with static loss weighting approaches. In the static variant, the balance between style and content loss remains constant throughout training. As shown in Table 3, progressively transitioning from low-level detail emphasis to high-level structural coherence yields significantly better results. Specifically, the dynamic strategy outperforms the static approach by improving structural integrity (DINO score improved from 0.608 to 0.629) and style fidelity (DS improved from 0.584 to 0.567), demonstrating that adjusting the training objective adaptively is critical for effectively capturing both detailed style textures and robust content structures.

**Table 3:** Ablation study on the progressive loss transition strategy.

Loss Strategy	DS ↓	DINO ↑	CLIP ↑
Static	0.584	0.608	0.652
Progressive (Ours)	**0.567**	**0.629**	**0.659**

**Fig 5:**
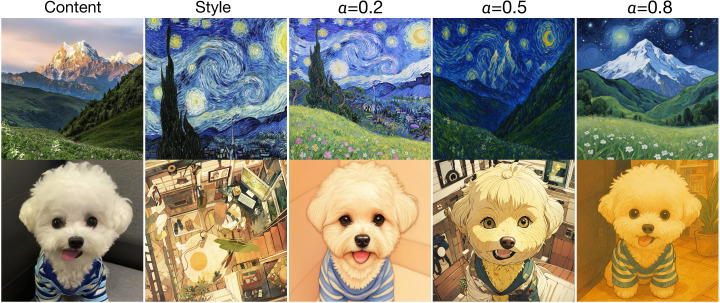
Visualization Results of Different α. Reprinted from [https://pan.baidu.com/s/10_CjlBAaXZ6vB_RONzfU3g?pwd=b97i] under a CC BY license, with permission from Yi Yang, original copyright 2025.

**Influence of Decoupled Inference Controller.** Finally, we assess the impact of our proposed Decoupled Inference Controller by comparing inference performance with and without dynamic content-style interpolation capabilities. Without DIC, the inference is performed using fixed, averaged module weights. As indicated in Table 4, incorporating DIC substantially improves both quantitative scores and user satisfaction. The flexibility provided by DIC enhances the model’s adaptability to diverse stylization intensities, significantly reducing content misalignment (DS improved from 0.579 to 0.567) and increasing style coherence (CLIP improved from 0.648 to 0.659). User preference tests also strongly favor the dynamic approach, demonstrating the practical value of adaptive inference control. Furthermore, we conduct an ablation study on the influence of the scaling factor α in Fig 5. We observe that when α is small, the generated images exhibit stronger stylistic characteristics, whereas larger values of α lead to better preservation of content information. These findings are consistent with our expectations and support the effectiveness of α in balancing style and content fidelity.

**Table 4:** Ablation study on the Decoupled Inference Controller.

Inference Method	DS ↓	CLIP ↑	User Preference ↑
Without DIC	0.579	0.648	64%
With DIC (Ours)	**0.567**	**0.659**	**83%**

Collectively, these ablation studies clearly demonstrate that each component in UltraStyle contributes significantly to achieving superior performance in high-quality style transfer, justifying our proposed design choices.

## Conclusion

In this paper, we introduced UltraStyle, a novel diffusion-based style transfer framework enhanced by a dual-phase training strategy, a progressive loss transition mechanism, and a Decoupled Inference Controller. By addressing the fundamental issues of feature entanglement, content leakage, and structural distortions that commonly plague existing methods, UltraStyle provides significant improvements in both style alignment and content preservation. Comprehensive qualitative and quantitative experiments consistently demonstrate our method’s superior performance over leading baselines such as StyleAligned, StyleID, and B-LoRA. Detailed ablation studies further validated the individual effectiveness and collective contributions of each key component in UltraStyle. Specifically, dual-phase training was shown to effectively reduce feature entanglement, the progressive loss transition significantly enhanced the balance between detail preservation and structural coherence, and the DIC provided crucial adaptive control during inference, leading to improved user satisfaction and performance metrics. Looking forward, several promising avenues exist for further research. Integrating UltraStyle with advanced generative paradigms such as transformer-based diffusion models may further enhance the robustness and diversity of generated stylizations. Additionally, exploring more sophisticated adaptive inference strategies capable of region-specific or temporally coherent stylization represents a valuable direction, particularly for dynamic content such as videos. We anticipate that UltraStyle will serve as a foundational framework, enabling continued innovation in style transfer and broader generative applications.
